# Dramatic action: A theater-based paradigm for analyzing human interactions

**DOI:** 10.1371/journal.pone.0193404

**Published:** 2018-03-08

**Authors:** Yuvalal Liron, Noa Raindel, Uri Alon

**Affiliations:** 1 Theater lab, Weizmann Institute of Science, Rehovot, Israel; 2 Dept. Molecular Cell Biology, Weizmann Institute of Science, Rehovot, Israel; Danmarks Tekniske Universitet, DENMARK

## Abstract

Existing approaches to describe social interactions consider emotional states or use ad-hoc descriptors for microanalysis of interactions. Such descriptors are different in each context thereby limiting comparisons, and can also mix facets of meaning such as emotional states, short term tactics and long-term goals. To develop a systematic set of concepts for second-by-second social interactions, we suggest a complementary approach based on practices employed in theater. Theater uses the concept of dramatic action, the effort that one makes to change the psychological state of another. Unlike states (e.g. emotions), dramatic actions aim to change states; unlike long-term goals or motivations, dramatic actions can last seconds. We defined a set of 22 basic dramatic action verbs using a lexical approach, such as ‘to threaten’–the effort to incite fear, and ‘to encourage’–the effort to inspire hope or confidence. We developed a set of visual cartoon stimuli for these basic dramatic actions, and find that people can reliably and reproducibly assign dramatic action verbs to these stimuli. We show that each dramatic action can be carried out with different emotions, indicating that the two constructs are distinct. We characterized a principal valence axis of dramatic actions. Finally, we re-analyzed three widely-used interaction coding systems in terms of dramatic actions, to suggest that dramatic actions might serve as a common vocabulary across research contexts. This study thus operationalizes and tests dramatic action as a potentially useful concept for research on social interaction, and in particular on influence tactics.

## Introduction

Defining the facets of human social interaction is central to fields ranging from psychology and sociology to artificial intelligence and human-machine interface. Much research is focused, for example, on recognition and classification of human states which play a role in social interactions, such as emotions\. The classic studies of Ekman[1–3] on basic emotions such as anger, sadness, fear, and happiness has led to work in computer science and psychology on the recognition and elicitation of emotion in diverse stimuli[4–14]. In addition to emotion, other well-known facets of human states and behaviors include motivation[15,16], narratives[17], speech acts[18] and other constructs.

Whereas adequate concepts exist to characterize human individual states, there is a lack of concepts to characterize the fundamentally dyadic nature of social interactions, and especially second-by-second influence tactics that people exhibit when they interact with each other. This lack of concepts is evident in calls to consider cognitive processes in the ‘we-mode’[19] and in the emergence of fields such as social neuroscience[20]. Existing concepts for social interaction seem to be context-specific. Detailed studies of dyadic interaction in contexts such as parent-infant[21], therapist-client[22], doctor-patient[23,24] and married couples[25,26] use ad-hoc descriptors to analyze second-by-second interactions. For example, analysis of married couples can predict divorce rates based on interactions described as contempt, stonewalling, criticism and defensiveness[26]. Client-therapist relations are improved by positive regard and attending[22], and placebo effects are increased by appropriate shifts from engaged listening to high-status suggestion[27]. Other approaches analyze body language [28,29] or speech prosody[30]. Although useful in their specific contexts, there is a lack of a systematic set of concepts that capture what people do in dyadic social interactions on the timescale of seconds.

To approach such a systematic set of concepts, we consider an arena that has developed powerful approaches to re-create social interaction: the practice of theater (including cinema and other modes of performance). Theater often aims to create specific portrayals of human interaction. Accumulated experience shows that instructions for actors based on individual psychological factors such as emotion, motivation and narrative are not enough to generate the desired performance[31]. Theater directors and actors rely on an additional layer which is thought to be essential for creating believable interaction. This facet of behavior is called dramatic action[32]. Here we aim to operationalize and test dramatic action as a potentially useful concept for research on social interaction, and in particular on influence tactics.

Dramatic action (DA) in theater is an informal concept that indicates what kind of effort the character makes in each short segment of interaction. DA can be defined as the effort that one makes to change the psychological state of another. Thus, DA is a fundamentally dyadic concept. Examples of DA are ‘to threaten’–an attempt to make the other frightened, and ‘to cheer’–an attempt to make the other happy.

DA was described qualitatively in the theater literature by Constantin Stanislavsky (who called it action)[31], Lee Strasberg (who developed the American Method, based on Stanislavsky's concept of actions)[33], Uta Hagen (who called it tactics)[34] and most explicitly by Ivana Chubak[32]. DA in acting "encourages performances with accurate and dramatic communication between characters"[35], and "enforces a specificity which can liberate the actor’s performance and ensure a cohesive integrated character with each moment leading naturally onto the next"[35]. Acting without DA "results only in the most disgusting artificiality"[31].

The concept of DA is a central element of the “Active Analysis” method, created by Stanislavsky as a practical method to research plays [36]. Lists of DAs for actors have been compiled including a thesaurus of dramatic actions[35]. However, the notion of DAs has not carried over to behavioral research, because it has yet to be quantitatively documented and categorized.

To complete the introduction, we summarize properties of dramatic actions from the theater literature. DAs are observable behaviors whose timescale is on the order of seconds. In this way, DAs differ from internal motivations[15,16], which last the entire play, and goals, which can last an entire scene. A character can change dramatic actions rapidly in an attempt to reach a goal. For example, in the Shakespeare play, Macbeth’s overall motivation is to gain power. In Act III Scene I, Macbeth's goal is to asses Banquo's loyalty and gather information. Macbeth's tactic is to befriend Banquo (using the DAs ‘to flatter’, ‘to empower’) and to pin the blame for Duncan's murder on others (using DAs ‘to inflame’, ‘to incite’).

DAs are distinct from emotions because they are not states but instead are the effort to change the other’s state. One can be happy, angry, or sad and still threaten someone else. Whereas emotions are adjectives, DAs can be described by transitive verbs that fit the template “I ____ you”. DAs are related to a subset of Austin’s concept of speech acts[18] called perlocutionary acts: utterances performed to affect the listener. Many DAs however, are not speech acts, and in fact do not require speech.

**Fig 1:**
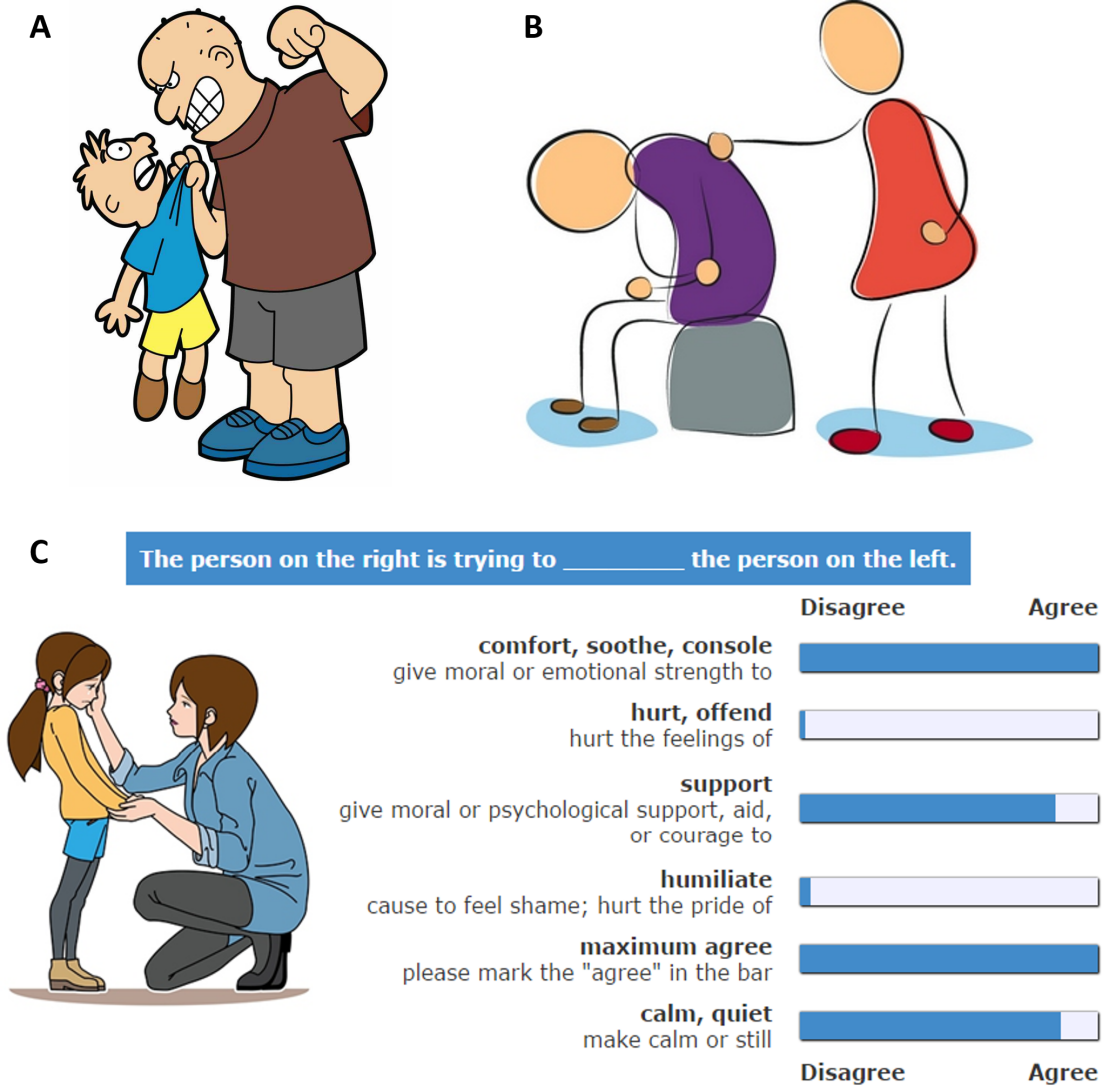
Examples of dramatic actions. (A) The character on the right is performing the DA ‘to threaten’. This DA seems to be successful because the other person in the image shows fear. (B) The DA performed by the person on the right is ‘to comfort’. Here the person receiving the action still seems sad, meaning that the action has not yet taken effect. This DA may or may not work in the future. (C) Schematic of the basic unit of the survey in experiment 1. Online participants used a mouse to set a value on each of the continuous slider-scales (thus there is no default agreement value). The DA words were taken from List C (Table 1) using a pseudo-random order. The definitions of the words were taken from WordNet. See Figure C in S1 File for the full screenshot and more details. Cartoons reprinted from Shutterstock.com under a CC BY license, with permission from Shutterstock.

We note that DAs need not necessarily succeed. Fig 1A illustrates a successful DA ‘to threaten’: the character on the left is afraid after being threatened by the character on the right. In contrast, Fig 1B shows an example of a DA, ‘to comfort’, that has still not changed the state of the other person; this DA may succeed in the future, be ignored, or lead to unexpected results. Regardless of success or failure, we can still detect the effort made to change the state of the other- the DA. Often, DAs are part of people’s habitual behavior, and can be performed without conscious deliberation.

Furthermore, the same text can be said with different DAs: For example, the text ‘come here’ can have a different DA if said by a parent soothing a child, or by a drill sergeant threatening a recruit. DAs are often conveyed through non-verbal signals including body-language and gestures, facial expressions, speech, and physical actions. DAs can be understood even if some of the signals are not perceived, such as visual stimuli without sound or movement (e.g. seeing an image) or auditory stimuli without vision (e.g. hearing someone on the phone). Even animals and babies can detect, carry out, and respond to dramatic actions[37]. Babies can activate their surrounding adults and react to soothing voices; dogs can try to cheer people around them or threaten other dogs.

Here, we attempt to operationalize the concept of DA, in order to test these subjective notions and provide a basis for quantitative research on DA. We categorize major groups of DAs and developed a preliminary set of stimuli to test whether people reliably agree on identifying these DAs. We then demonstrate how several commonly-used interaction coding systems can be interpreted in terms of DAs. For this purpose, we use methods from research on emotion categorization[3] and elicitation[38]. We hypothesize that

(i) language includes prevalent words that describe DAs (lexical hypothesis[39])
(ii) people can recognize DAs in images
(iii) people agree on which DA is seen
(iv) people perceive DAs in images as different from emotion

## Methods

### List of basic dramatic actions

We used the WordNet database[40] (version 3.1) to compile a list of unique verbs that fit the frame ‘somebody—-s somebody’, resulting in 2482 synsets (sets of synonymous words). The words from the synsets were merged into list A of 3602 verbs. We used the Google Ngram[41] database (latest year column from Ngram version 2) to sort list A by frequency of appearance in books. We chose 70 words of relatively high frequency that appear in a previous list of DAs from different sources (Appendix A in S1 File), 30 additional words that were judged as DAs by one of the authors (YL) based on theater-directing experience, as well as the synonyms of these chosen words from the WordNet database. We avoided conceptual words such as ‘to educate’, and metaphors for DAs such as ‘to crush’, seeking instead words that have direct meaning in terms of changing the others state. For example, ‘to crush’ could be ambiguous in the sense that it could mean ‘to dominate’, ‘to terrorize’, ‘to physically stress’, or ‘to humiliate’, in different contexts. This process resulted in list B of 150 DAs (Table A in S1 File). This list includes words that have overlap in meaning. For example, ‘hurry’, ‘rush’ and ‘urge’. To reduce list B to a minimal list which represents, with as few verbs as possible, large classes of dramatic actions, we grouped the DAs in list B using hierarchical relations defined by WordNet (synonyms, hypernyms), and presented them as a forest graph (collection of hierarchical trees, Figure A in S1 File). Collecting words from the main trees resulted in list C which contains 22 verbs, our preliminary suggestion for primary DA groups (Table 1).

**Table 1:** Categorized list of DA verbs used for experiment 1 (List C).

	Category	Action	definition
**Induce Emotion**	Sadness	hurt, offend	hurt the feelings of
		humiliate	cause to feel shame; hurt the pride of
		Upset	cause to lose one's composure
		Shame	cause to be ashamed
	Fear	frighten, scare	cause fear in
		Intimidate	make timid or fearful
		Bully	discourage or frighten with threats or a domineering manner; intimidate
		Threaten	pose a threat to; present a danger to
	Anger	Anger	make angry
	Happiness	Uplift	fill with high spirits; fill with optimism
		Encourage	inspire with confidence; give hope or courage to
		Cheer	cause (somebody) to feel happier or more cheerful)
		Support	give moral or psychological support, aid, or courage to
	Surprise	Surprise	cause to be surprised
	Disgust	repel, repulse	be repellent to; cause aversion in
**Change arousal**	Reduce arousal	comfort, soothe, console	give moral or emotional strength to
		calm, quiet	make calm or still
	Increase arousal	stimulate, energize	cause to be alert and energetic / raise to a higher energy level
		Urge	force or impel in an indicated direction
**Change status**	Increase own status	Impress	impress positively
	Lower other's status	Criticize	find fault with; express criticism of; point out real or perceived flaws
		Scold	censure severely or angrily

### Experiments design

#### Subjects

A total of 231 subjects participated in two experiments (experiment 1: 150 subjects, 63 women; experiment 2: 115 subjects, 57 women; 34 participated in both experiments). Experiments were performed on the Amazon Mechanical Turk (MTurk) platform. The online surveys were restricted to US residents, with a record of at least 1000 previously approved MTurk HITs (human intelligence tasks). All participants passed a short test for English comprehension. Participants were paid 6 US cents per HIT, up to a maximum of 90 HITs in experiment 1 and 60 HITs in experiment 2. Ethics approval was obtained specifically for the surveys in this study by the IRB of the Weizmann Institute of Science, Rehovot, Israel. Consent was not obtained since the surveys were answered anonymously online. All the data was analyzed anonymously.

#### Stimuli

We used a set of 30 images (Images were purchased from Shutterstock.com, see S1 Text for license information). The images were in minimalistic styles (cartoons, silhouette, contour drawing), and were balanced for gender (26 women out of 60 characters). Images had white background with the person to the right performing the DA, and were sized to fit in a 400x400 pixel box. In experiment 2, a black arrow was added next to one of the characters. 30 images were collected and used for the surveys (Figure B in S1 File). 3 images were removed from the analysis since they were horizontally flipped due to a coding error, for a final set of 27 images.

#### Ratings

The basic unit of the MTurk survey (a single HIT) was composed of a single cartoon stimulus and a list of 8 DA words selected from List D, together with their WordNet definitions (Figure C in S1 File). The subjects were asked to rate how well each DA completes the sentence “The person on the right is trying to _____ the person on the left” using a continuous agree-disagree horizontal slider scale, in which a mouse is used to set a value. There was no initial (default) value, in order to avoid biasing the subjects. The slider scale result was converted to a score between 0–100. In order to verify that the survey participants accurately read and answered the survey and did not randomly fill it, 2/3 of the HITs contained an “attention check” question instead of one of the DAs, asking the participant to mark either the “Agree” or “Disagree” in the bar. The DAs were arranged in pseudo-random order. We used the results from all subjects, regardless of the number of HITs they performed. Each HIT also included an option to type in a word to describe the image (free text). This data is not considered in the present study.

Experiment 2 used an identical design except that it was aimed at surveying emotions instead of DAs. Thus(i) the question posed is “The person on the right (left) is feeling _______.”, (ii) one of the two characters in the stimulus is marked by a black arrow and (iii) instead of 8 DA words, six emotion words (Happy, Sad, Angry, Afraid, Surprised, and Disgusted) were used without definitions (Figure D in S1 File).

### Data filtering and analysis

#### Data filtering

We removed data from respondents that did not meet the attention check in two or more questions (16% of respondents, total of 29% of questions removed).

#### Consistency analysis

We tested the consistency of the responses using the approach of Ref [42]. We divided the responses for each of the 594 image-word combination into two equal groups (averaging 21.3 workers per group) and compared median responses across groups. Correlation was 0.91, and the difference was not significant (n = 594, P = 0.9, two-tailed two-sample Kolmogorov-Smirnov test), showing that the procedure yields consistent responses.

#### Criterion for agreement

To compute agreement for each question (image-DA combination), one cannot use Krippendorff‘s alpha because it requires comparison between at least two questions [43]. We therefore used a statistical test that picked up on the fact that, for most of the questions, the majority of respondents agreed on high or low scores. The challenge was that the responses had a noisy structure: often a sizable minority of the respondents had a wide range of responses. To address this, we used a statistical test based on bootstrapping [44], keeping the statistics of each respondent the same, see (Appendix B in S1 File). For questions that passed the criterion for agreement, we used the median score.

#### Data analysis

**Fig 2:**
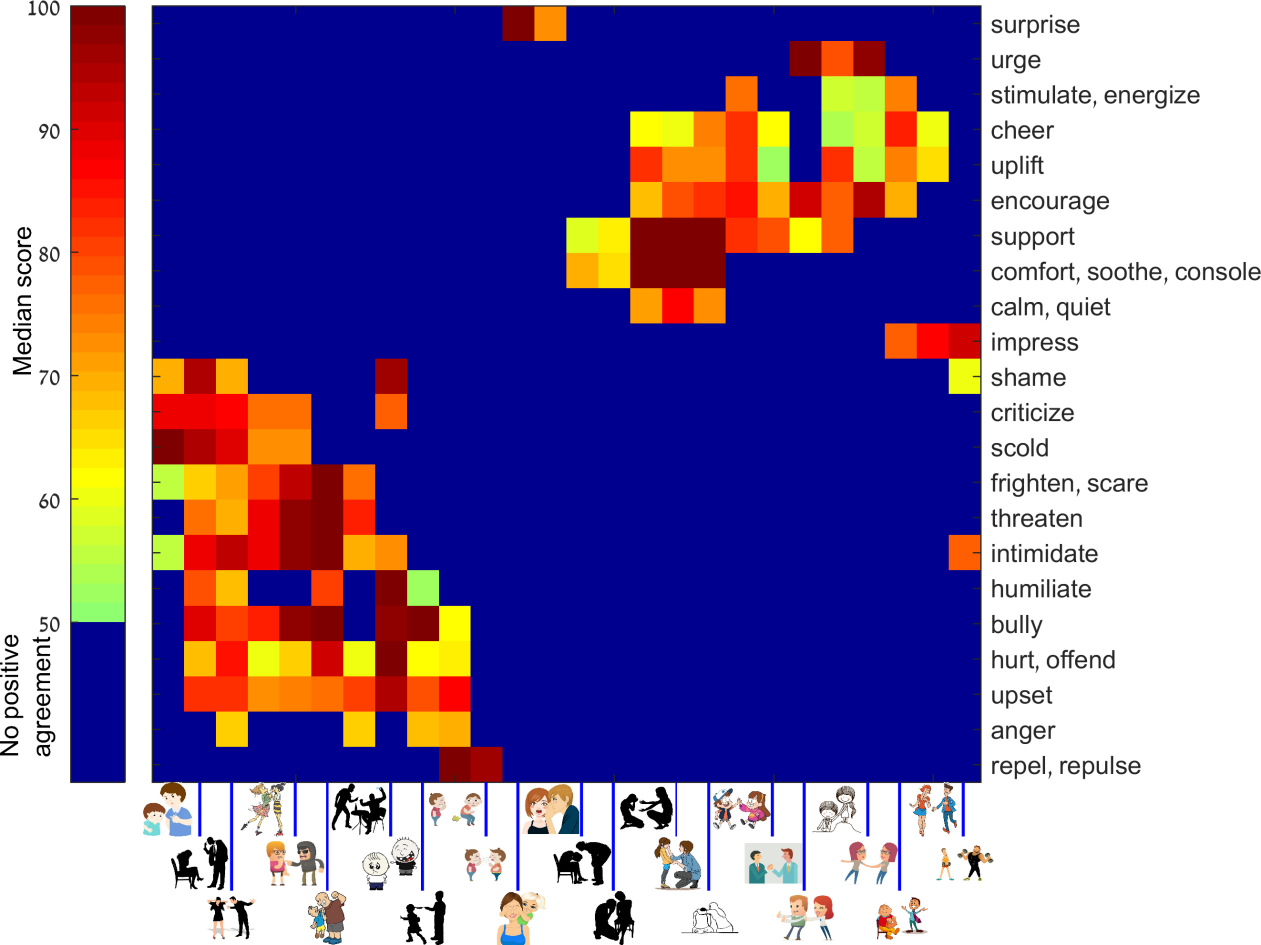
Images and verbs clustered into groups according to the raters’ agreement. Shown is the median score from 60 replies for each pair of images and DA verbs that exceed a statistical threshold (blue marks pairs below threshold). Images and verbs were ordered according to clustering, such that images that are close to each other have similar DA verbs, and DA verbs that are close to each other have similar images. The lower left block describes negative valence DAs, and the top right block represents positive valence DAs. Cartoons reprinted from Shutterstock.com under a CC BY license, with permission from Shutterstock.

We clustered the responses (Fig 2) using the clustergram function of MATLAB v2015b with correlation distance (RowPDist, ColumnPDist = 'correlation'). For principal component analysis (PCA) we used the pca function of MATLAB v2015b with default settings. A second PCA analysis was done after splitting the words into two distinct groups, defined by the sign of the first PC (positive and negative valence). The analysis method of experiments 1 and 2 was identical (Figures H-I in S1 File).

## Results

### List of primary DA groups

To define a list of dramatic actions, we used the lexical hypothesis[39], that language should contain common words that describe important characteristics of human behavior. To adapt the lexical hypothesis to dramatic action, we note that DA is the effort to change the psychological state (e.g. emotion, status, and energy level) of another. Words for DAs are therefore transitive verbs, verbs in which an agent acts on someone else.

We formed a list of unique verbs that fit the frame ‘somebody—-s somebody’ using the WordNet database[40], and retained words of high frequency using Google Ngram[41]. We find that these words fall into three categories: attempts to change emotions, to change energy level and to change status. Removing synonyms, we end up with a list of 22 verbs, our preliminary suggestion for primary DAs (Table 1, see Methods for details).

### Stimuli set of cartoon images for DAs

**Fig 3:**
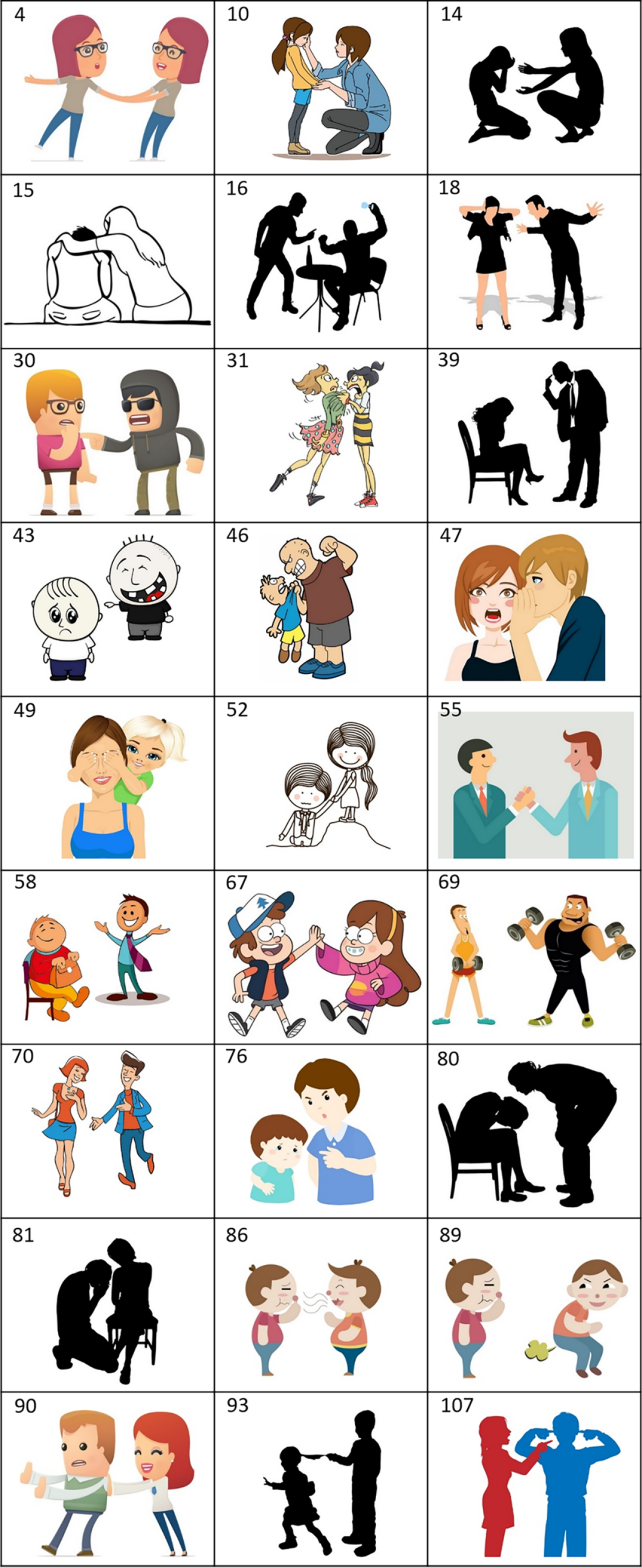
The DA stimuli set used in the experimental analysis. Reprinted from Shutterstock.com under a CC BY license, with permission from Shutterstock.

We next asked whether people can identify and agree on DAs in defined stimuli. For this purpose, we used the list of 22 DA verbs to choose evocative images from online image databases. The images show two people with one performing a clear DA on the other. In order to reduce the potential for extraneous information, we chose minimalistic cartoons with no text. We selected several styles of cartoons including silhouettes, contour drawings and cliparts, aiming to avoid biases of age and gender. The set of 27 visual stimuli is shown in Fig 3.

### Survey of DA words to describe the cartoon stimuli

To study how people describe the cartoon stimuli, we used Amazon Mechanical Turk[45]. In experiment 1, participants (N = 150, after data filtering N = 126, see methods) filled out a survey composed of units. Each unit showed a single cartoon stimulus and a list of 8 DAs, with their definitions (Fig 1C). The subjects were asked to rate how well each DA verb completes the sentence: “The person on the right is trying to _____ the person on the left”, using a continuous agree-disagree slider scale with no default value.

Each of the 27 cartoons was rated for each of the 22 DA words 39–47 times (total of 28137 answers). Responses tended to be dichotomous (Figure E in S1 File), with ~62% scoring below 10 and ~10% scoring above 90, compared to 2.7%-3.9% of the responses in the other 8 decile bins (bootstrapping p<10^−4^).

**Fig 4:**
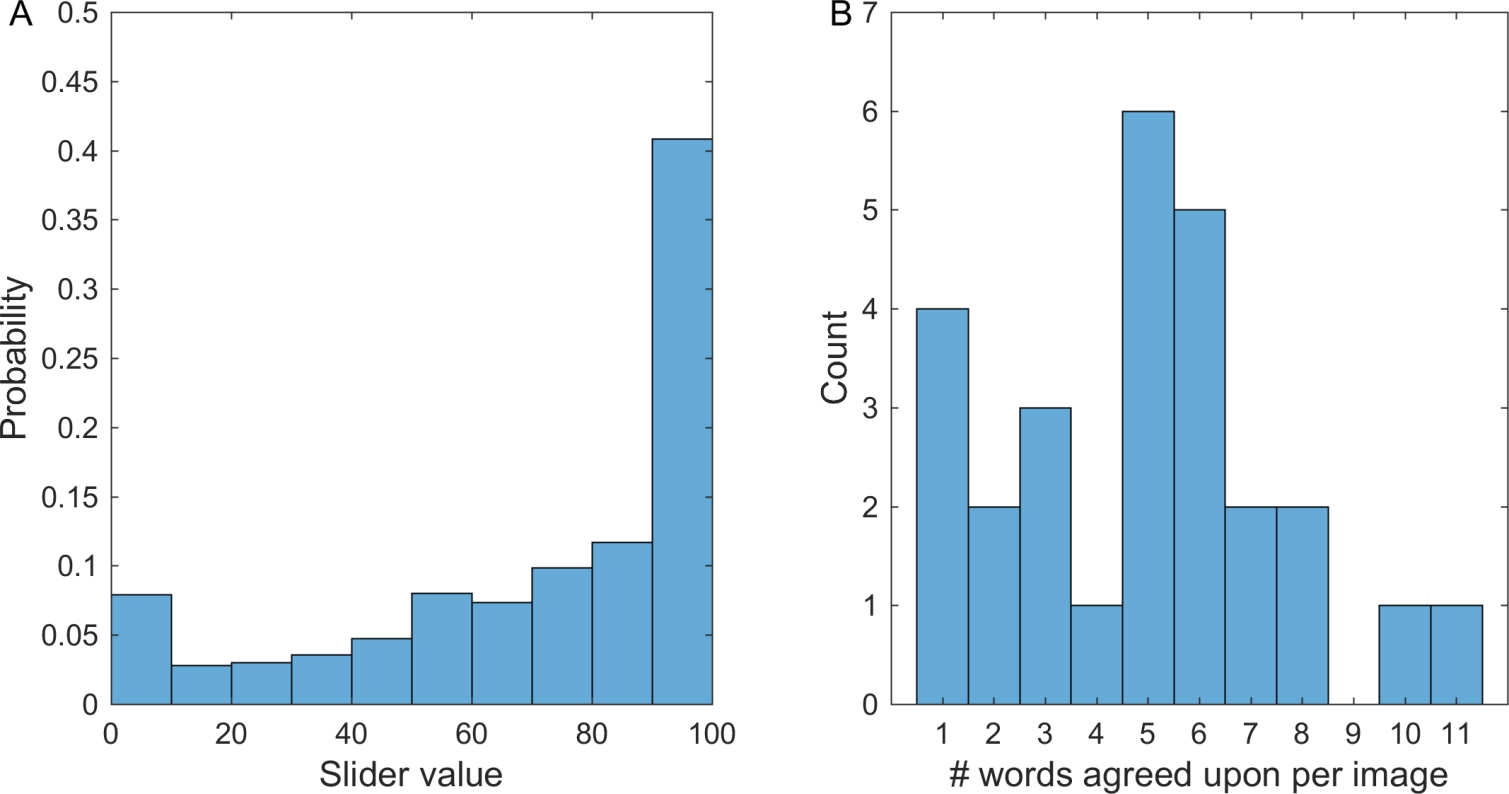
(A) Distribution of all answers to high-score-agreement questions of survey 1. (B) The distribution of high-score-agreement DA words per image. For example, two images had 7 high-score-agreement DA words.

Overall, the survey showed excellent inter-rater consistency (Methods). We also computed inter-rater agreement for each question–each pair of DA verb and stimulus (see Methods). We find that 71% of the survey questions showed significant inter-rater agreement (p<10^−4^), both when raters agreed on a high score for a DA, or on a low score. Median scores exceeded 90 in a sizable fraction of the responses (23%). The distribution of the responses for questions with agreement on high scores is presented in Fig 4A. One cartoon (#107) got no high-score agreement on any DA word, and was removed from further analysis. All of the other cartoons showed between 1 and 11 high-score-agreement DA words out of the 22 presented, with a median of 5 DA words per image (Fig 4B). Several cartoons were quite specific and showed 1 DA verb (for example: image #47, surprise; image #86, repel) or 2 DA verbs (images #80, #81, comfort and support).

We conclude that people agree on DA words to describe the cartoon stimuli.

### Valence and DA classification

We next asked whether DAs can be grouped according to similarity in the responses. For this purpose, we analyzed the structure of the response data using a clustering approach (see Methods). Clustering separates the data into groups of images with similar responses, and groups of responses with similar images. Clustering showed two clear groups of DAs and two corresponding groups of cartoon stimuli (Fig 2). The groups can be interpreted as DAs with positive and negative valence (e.g. ‘support’ vs ‘humiliate’).

To gain finer resolution on this gradation, we employed a method used to analyze the structure of emotional elicitation of images[38]. We reduced the dimension of the data using principal component analysis (PCA) on the response matrix. We considered each cartoon as a point in a 22-dimensional space whose axes are the responses to the 22 DA words (a value between 0–100 for each coordinate). We find that the first two PCs account for 59% of the variation (p<10^−4^ compared to 10000 shuffled datasets), suggesting that two axes describe the data well.

**Fig 5:**
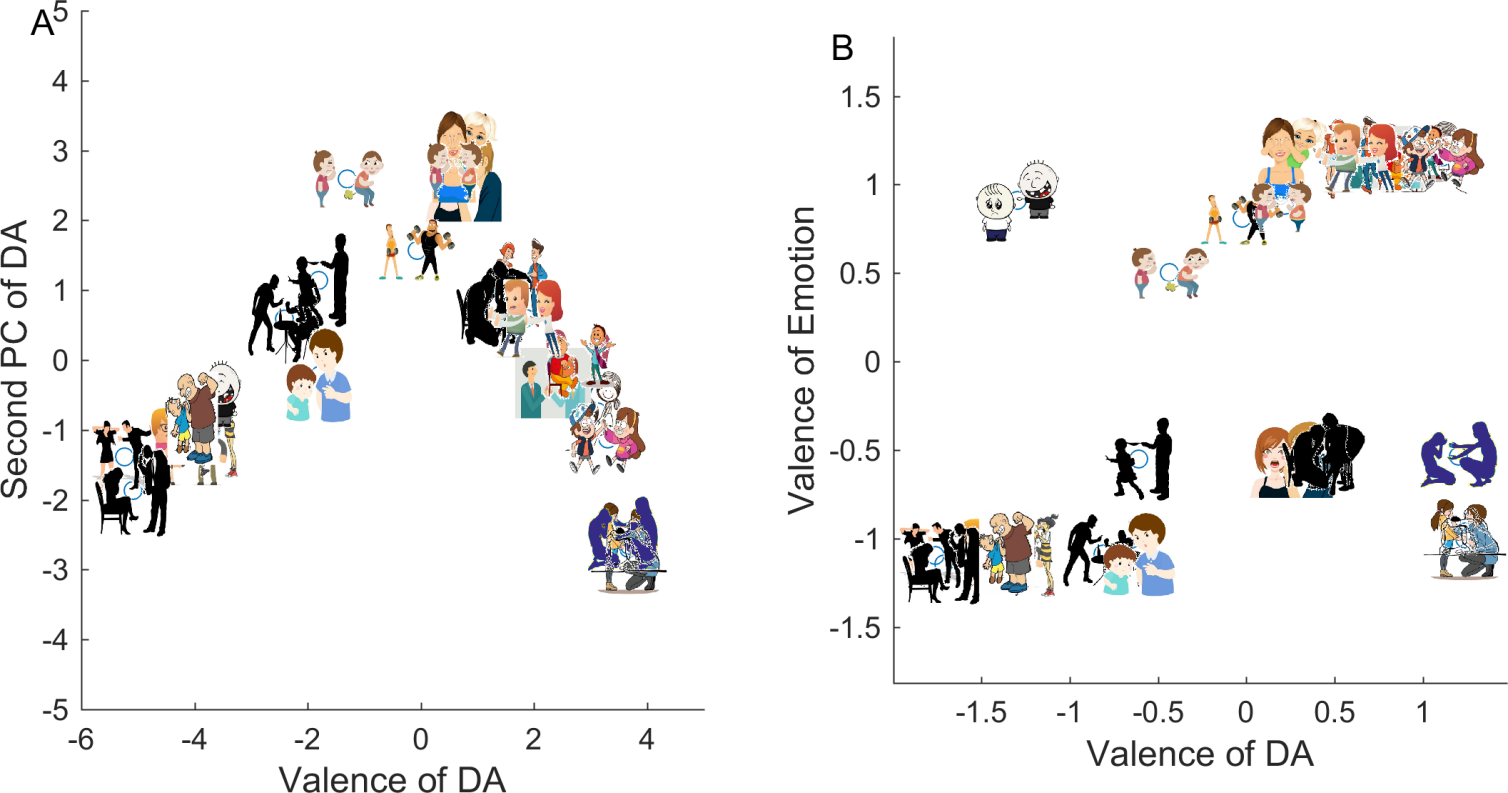
(A) Cartoon stimuli in the space of the first two principal components (PCs) show a V-shape. Each cartoon was described as a vector of responses in a 22-dimensional space of the DA words and projected on the PC1-PC2 plane. (B) Valence of the DA and the valence of the emotion of the actor are not identical. Cartoon stimuli organized by PC1 of DA and PC1 of emotions. Cartoons that didn't receive significant agreement in either DA labels or emotion labels were not included. There is a moderate correlation between the PCs (r = 0.49, p = 0.01). However, the cases where valence of the two PCs is opposite are not outliers, but instead are valid sub groups of the stimuli set. Reprinted from Shutterstock.com under a CC BY license, with permission from Shutterstock.

Plotting the stimuli in the space of these axes results in a distinctive V-shape (Fig 5A). This shape is reminiscent of V-shapes found in PCA of photo stimuli sets tested for eliciting emotion words[38]. In the field of emotion elicitation, the two axes are interpreted as emotion valence and arousal. This interpretation seems to apply to the DA as well. Analysis of the first PC suggests that it corresponds to the valence of the DAs. At one extreme of loadings are the words ‘support’, ‘encourage’, ‘uplift’, ‘cheer’, and at the other extreme are ‘bully’, ‘intimidate’, ‘upset’, and ‘hurt’. The second PC is less easy to interpret, but at least among the positive valence DAs, can be interpreted as arousal (extreme words ‘comfort’, ‘calm’, and ‘support’ versus ‘stimulate’, ‘impress’, ‘urge’).

### Dramatic actions and emotions

Axes of valence and arousal seem to be found in both DA and in studies that classify emotions[38]. This raises the question whether DAs are distinct from emotions. One might argue that the responses for DA words actually register the emotion of the figures in the cartoon, rather than a distinct construct.

To test the similarity between DAs and emotions, in experiment 2 we repeated the survey with the same stimuli, but asked participants to describe the cartoons with six Ekman basic emotions instead of the DA words. Participants were asked to describe the emotion of the character on the right, which is the character performing the DA. Below we also describe results in which participants were asked to describe the emotion of the character on the left, which is the character receiving the DA.

We find that people agree on emotion words to describe 69% of the stimuli (p<10^−4^, compared to 10000 shuffled datasets). For 5 stimuli, there was no significant agreement (#14, #47, #80, #81 and #93). The first PC of the emotion responses is valence in accordance with previous studies on emotion elicitation[38].

**Fig 6:**
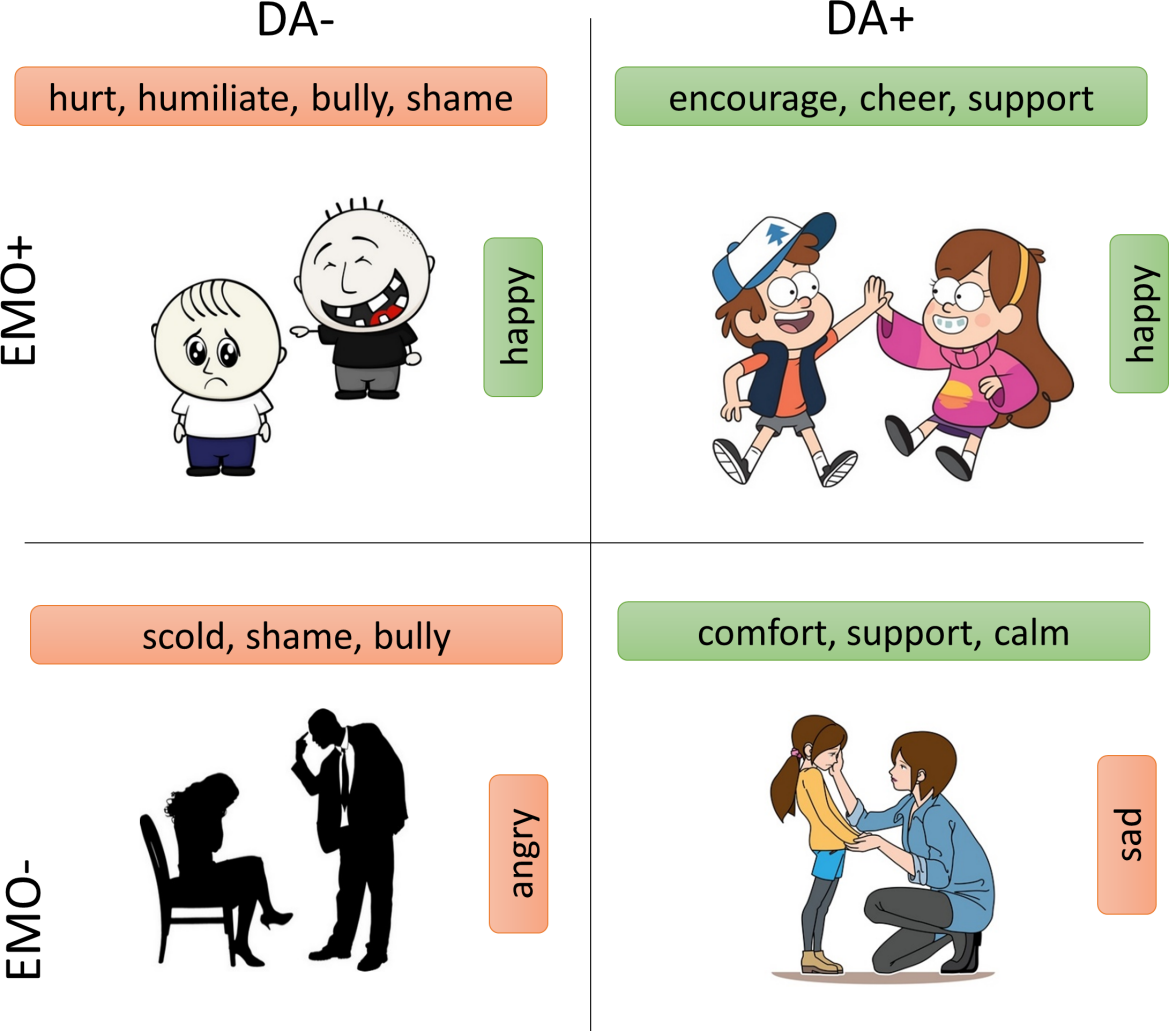
Examples of stimuli where the DA valence is not correlated to emotion valence. The same DA, ‘to support’, can be performed while being either happy or sad (right side of the image). Additionally, one can perform a negative DA such as ‘to bully’ while being either happy or angry. Inter-rater agreement in all cases was very high (median>64, p<10–4). Cartoons reprinted from Shutterstock.com under a CC BY license, with permission from Shutterstock.

Most importantly, this survey allowed us to ask whether the valence of a cartoon according to DA words matches the valence according to emotion words. We find that the valence of DA and emotion are correlated to a medium extent (r = 0.49, p = 0.01, Fig 5B). As expected, negative valence DA often comes with negative valence emotion of the actor. However, this correlation is not absolute. In about 30% of the cartoons, the valence of the DA and the emotion were opposite, with high inter-rater agreement (p<10^−4^). For example, a negative DA such as ‘to hurt’ comes with a negative emotion (anger, cartoon #46), or a positive emotion (happiness, cartoon #43) in the person who is doing the hurting. Likewise, a positive DA such as ‘to support’ can come with a positive emotion (happiness, cartoon #52) or a negative emotion (sadness, cartoon #15) in the person who is doing the supporting. Representative cases in which different emotions are found for the same DA are shown in Fig 6.

This highlights the difference between emotion, a state of the person, and dramatic action, the effort to change the state of another.

In addition to testing the emotion of the person doing the DA, we also asked participants to choose emotion words for the person receiving the DA in a separate trial. Again, we find strong agreement on emotion words (median>50, p<10^−4^) except for a single cartoon (image #52). In about 50% of the cases the valence of the emotion and DA match, which we interpret as an image showing the characters after the DA took effect (e.g. bully took effect making the subject afraid, cartoon #46). In about 25% of the cases, the valence was opposite, which we interpret as an image before the DA took effect, or where the DA failed. For example, the recipient of a ‘to cheer’ DA can be shown as sad (before, cartoon #15) or happy (after, cartoon #58). The remaining 25% showed a weak valence signal and could not be interpreted.

### DAs and social interaction coding schemes

To connect the concept of dramatic action with the tradition of social interaction coding, we considered three widely-used coding systems from different research contexts. These are the Gottman system used to study interactions between married couples (SPAFF) based on videos[26], the FAU AIBO annotations used to develop emotion recognition algorithms based on auditory data of children commanding a robotic dog[46,47], and the Ambady system for rating doctor-patient interactions based on brief garbled audio samples[24]. In Tables B-D in S1 File we analyze each item of these coding systems in terms of three levels: goals, defined as the intent to achieve a behavior or attitude in the other, states such as emotion or energy levels, and tactics defined as short-term behaviors to achieve the goal, carried out by dramatic actions.

We find that most (about 80%) of the items in the Gottman and Ambady systems can be defined in terms of dramatic action. For example, the SPAFF code “contempt” is defined in terms of dramatic actions ‘to hurt’, ‘to belittle’ or ‘to humiliate’. The doctor-patient rating "sympathetic" is defined by the dramatic actions such as ‘to comfort’, ‘to soothe’, ‘to console’.

The rest of the items in the Gottman and Ambady systems are better defined as goals or states than as dramatic actions. For example, the SPAFF code “disgust” is an emotional state (described as involuntary). The code “defensiveness” is defined as a goal to deflect blame or responsibility. It can be sharpened by noting the dramatic actions used to achieve this goal. This distinction highlights at least two tactics for defensiveness mentioned in the SPAFF manual (i) lowering own status as a tactic, with the dramatic action of ‘to beg’, (ii) counterattacking as a tactic, with the dramatic actions of ‘to threaten’ or ‘to belittle’. We conclude that the concepts described here can help refine coding systems, and to disentangle the three levels of state, DA (tactic) and goal.

The coding of the FAU AIBO database, defined for detecting emotions from audio recordings, has 6 items that are better defined as states (emotions) than as dramatic actions. However, 3 other items are regarded by Batliner et al. as social interactions[47]. We find that these interaction-oriented items (motherese, reprimanding and emphatic) correspond to dramatic actions. The FAU AIBO annotation "motherese" can be defined in terms of dramatic actions as ‘to encourage’ or ‘to support’. Interestingly, these three interaction-oriented codes account for 96% of the non-neutral labeled words in the FAU AIBO corpus.

## Discussion

This study presents dramatic action (DA) as a concept for social interaction based on practice in theater. We define DA as the effort to change the state of another. We developed a list of DAs based on the lexical hypothesis, and a set of visual cartoon stimuli for the main DA classes. We found that people agree on DA words to describe the cartoons. The survey responses tend to be dichotomous (see figure E in S1 File). This can provide insight into the way people perceive DAs, that is, as binaries (present or not present). Moreover, people distinguish between the emotions of the characters in the cartoon and the dramatic action they are carrying out, showing that DAs and emotions are distinct constructs. DAs have a principal component based on human perception that can be described as valence. Finally, DAs can be used to interpret coding systems for social interactions in different contexts, and to disentangle the facets of goal, state and DA, suggesting that DAs can act as an analytic and unifying concept.

One can categorize the DAs according to the state they intend to change in the other: emotion, arousal or status. Some DAs attempt to elicit an emotion in the other: ‘to threaten’ elicits fear, ‘to hurt’ elicits sadness, and ‘to cheer’ elicits happiness. Other DAs attempt to change arousal in the other: ‘to soothe’ acts to reduce arousal, ‘to energize’ acts to increase it. Some DAs can be interpreted as the attempt to change the status of the other, possibly together with eliciting an emotion, such as ‘to insult’/’to humiliate’ which lowers the other’s status. DAs such as ‘to impress’ attempt to raise the actor’s status in the eyes of the other character. The present study can be expanded to include additional classes of DAs, such as DAs in which the actor has low status. For example, the DA ‘to beg’ elicits pity from the other; DA such as ‘to flatter’ attempts to raise the status of the other.

We find that a two-dimensional PCA mapping explains most of the variance in the current DA responses. To interpret the PC axes, we compared the mapping to the three dimensions suggested by Russell for emotions: valence, arousal and dominance. The valence dimension was helpful in separating DAs, and the arousal dimension may correspond to the second PC in positive DAs. We believe that the third dimension, dominance, is not evident in the present study because of the set of DAs that we chose. This set lacks an extensive test of the dimension of dominance, because it is missing, for example, DAs that raise the status of the other such as ‘to beg’ (lowering one’s own dominance), or ‘to flatter’ (raising the others dominance). Instead, all of the DAs related to power in the current study were DAs with negative valence that lower the others status, such as ‘to threaten’ and ‘to bully’. The dominance dimension may thus be included in the valence dimension in the present study. This analysis points for a way that future studies can explore Russell’s three dimensions, by adding DAs that more widely explore status/dominance relations.

A skeptic might say that dramatic actions are just another way to speak about the emotion of the person carrying out the DA. Indeed, both DAs and emotions seem to have a principal axis of valence. Moreover, it is natural to couple emotions like happy with DAs like ‘to cheer’ or emotions like anger with DAs like ‘to hurt’ or ‘to intimidate’. We therefore tested how people perceive the emotion of a character and the dramatic action that the character carries out. While some correlation between perceived emotion and DA was observed, we documented clear cases in which the same DA can be carried out with different, even opposite, emotions. For example, the DA ‘to cheer’ can be carried out by a sad or a happy person in different cartoons. The DA ‘to bully’ can be carried out by an angry or a happy person. We conclude that DA is a distinct layer for describing social interaction. It describes the effort to change the state of the other rather than describing the state itself.

Our results suggest that people use at least two classes of dramatic actions as influence tactics: one in the context of cooperation and one in the context of competition. In the context of cooperation, we identify the effort to help the other regulate negative emotions emanating from two independent emotion-regulation systems[48]. That is, some tactics (e.g., ‘to sooth’) are meant to decrease negative affect from negative high-arousal to positive low-arousal, such as ‘relaxed’), whereas other tactics (e.g., ‘to cheer’) are meant to increase positive affect from negative low-arousal to positive high-arousal, such as ‘happy’).

In the context of competition, the dramatic actions studied here include the effort to dominate the other. Additional dramatic actions, which can be addressed in future studies, can act to appease the other with ingratiation, apologies, etc.[49]. A final set of DAs can involve changing the context from competition to cooperation or vice-versa. Such DAs require subtlety going beyond the present stimuli, as in work on innuendos[50].

Dramatic action may help form a set of concepts for describing social interactions across research contexts. To demonstrate this, in Tables B-D in S1 File we suggest the relationship between dramatic actions and the descriptors in three interaction coding systems. It is evident that many of the codes match dramatic actions. Other codes correspond to goals (such as the intent to change behavior, attitudes) or states (emotional states such as disgust, sadness). The concept of dramatic action can help to disentangle these facets, and to sharpen the codes. The use of dramatic action can potentially reduce a nuisance encountered in some contexts, such as datasets for emotion recognition, where a large number of interactions are scored as neutral[30], presumably because the facet of DA is not captured by emotion codes.

This study used an unusual approach of adapting concepts from theater to a scientific endeavor. Theater was suggested as a model for studying human behavior by Perlin and Goldberg[51], Busso and Narayanan[52], Douglas-Cowie et al. [53] and Goffman[54]. An example of the application of theater approaches to study social interactions employed the mirror game as an experimentally and mathematically accessible model of joint improvisation[55–58], with applications to assessing attachment style[59] and for rehabilitation[60–62]. Theater approaches were also used to create specific scenarios in order to test the impact of doctors performance on the placebo effect[63]. Finally, theatrical improvisation was used to build a database[64] for study of human expressive behavior in dyadic interaction.

Limitations of this study include the use of a particular set of visual stimuli and DA words. The cartoons used here are biased towards extreme portrayals of the DA. Use of additional visual stimuli for DA can test the generalizability of the results. We used a single language (English) and a single country (US online participants), and the cultural aspects of DA thus remain to be studied. We used only static images- the use of motion as in short video clips[65,66] might increase the perception of DAs. Finally, we recognized that many subtle DAs go beyond the current study, for example the DA ‘to impress’ has subtypes such as to impress by wit, to impress by physical prowess and even to impress by one’s humbleness. A categorization of DAs may be too coarse to fully describe the continuum of the ways people act to change each other’s states, as well as the individuality and non-repeatability in which different people in different situations carry out DAs.

Future work can refine our understanding of DAs and how people carry them out. We believe that gaining literacy in DAs can be a form of emotional intelligence that can help people name what is going on in a communication, especially when presented with negative DAs. A good grasp of DA can help researchers elicit desired states in people, advancing fields such as emotion elicitation[38] and emotional body language[29]. It would be interesting to study how DAs can be synthesized in human-computer interactions. Such synthesis can be used to provide more human-like speech and action in human-computer interfaces. It is also of interest to study whether DA classification can be automated in order to analyze social interactions. Finally, the DA concept can provide a framework for research in psychology and neurobiology for understanding how human brains and bodies act in a coordinated way in order to affect another’s state, and how these actions are perceived.

## Supporting information

S1 File Supporting information file. Supporting Information for methods and data analysis.
(PDF)

S1 Text Figures permission. Information regarding the figures’ copyrights.
(PDF)
