# Supplementary material for: Dramatic action: A theater-based paradigm for analyzing human interactions
Source: PLoS One. 2018 Mar 8;13(3):e0193404. doi: 10.1371/journal.pone.0193404 (PMC5843267; doi:10.1371/journal.pone.0193404)
Supplement: S1 File — Supporting Information for methods and data analysis. (PDF) [file pone.0193404.s001.pdf]

The structure of influence tactics: a cartoon-based measurement system derived from the theater concept of dramatic action

Yuvalal Liron, Noa Raindel, Uri Alon

Supplementary Information

Table of Contents

Figures 2

A. Tree representation of DA words 2

B. All survey images 3

C. Screenshot of the survey 1 4

D. Screenshot of the survey 2 5

E. Distribution of all answers of survey 1 (DA) 6

F. Criteria to determine agreement is based on area under the cumulative distribution curve 7

G. Cumulative distribution of all high agreement questions of survey 1 8

H. Distribution of all answers of survey 2 (emotions) 9

I. Cumulative distribution of all high agreement questions of survey 2 10

Tables 11

Table A – List B – 150 DA words 11

Table B – Analysis of the Specific Affect Coding System (SPAFF) from Ref (1) 13

Table C – Analysis of the FAU emotion corpus annotations from Ref (3) 16

Table D – Analysis of ratings used in surgeons' voice-tone rating from Ref (4) 18

List of Appendices 20

A. Collection of Dramatic action (DA) lists 20

B. Criteria for inter-rater agreement 20

References 22

Figures

A. Tree representation of DA words

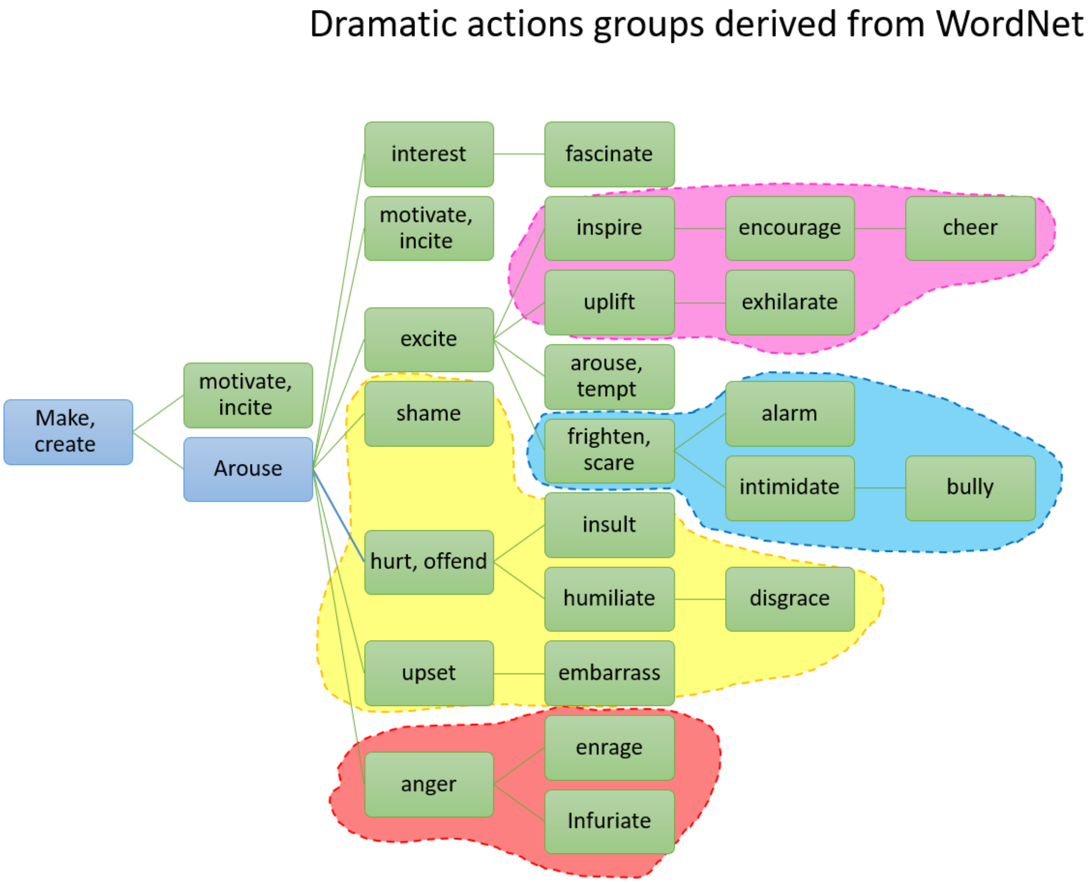

Figure A – Tree representation of DA words. The tree in this diagram is an example of one tree from a graph forest representation of the words in List B. The graph hierarchy s based on the WordNet relations of hyponym/hypernym, for example insult and humiliate are hyponyms of hurt.

B. All survey images

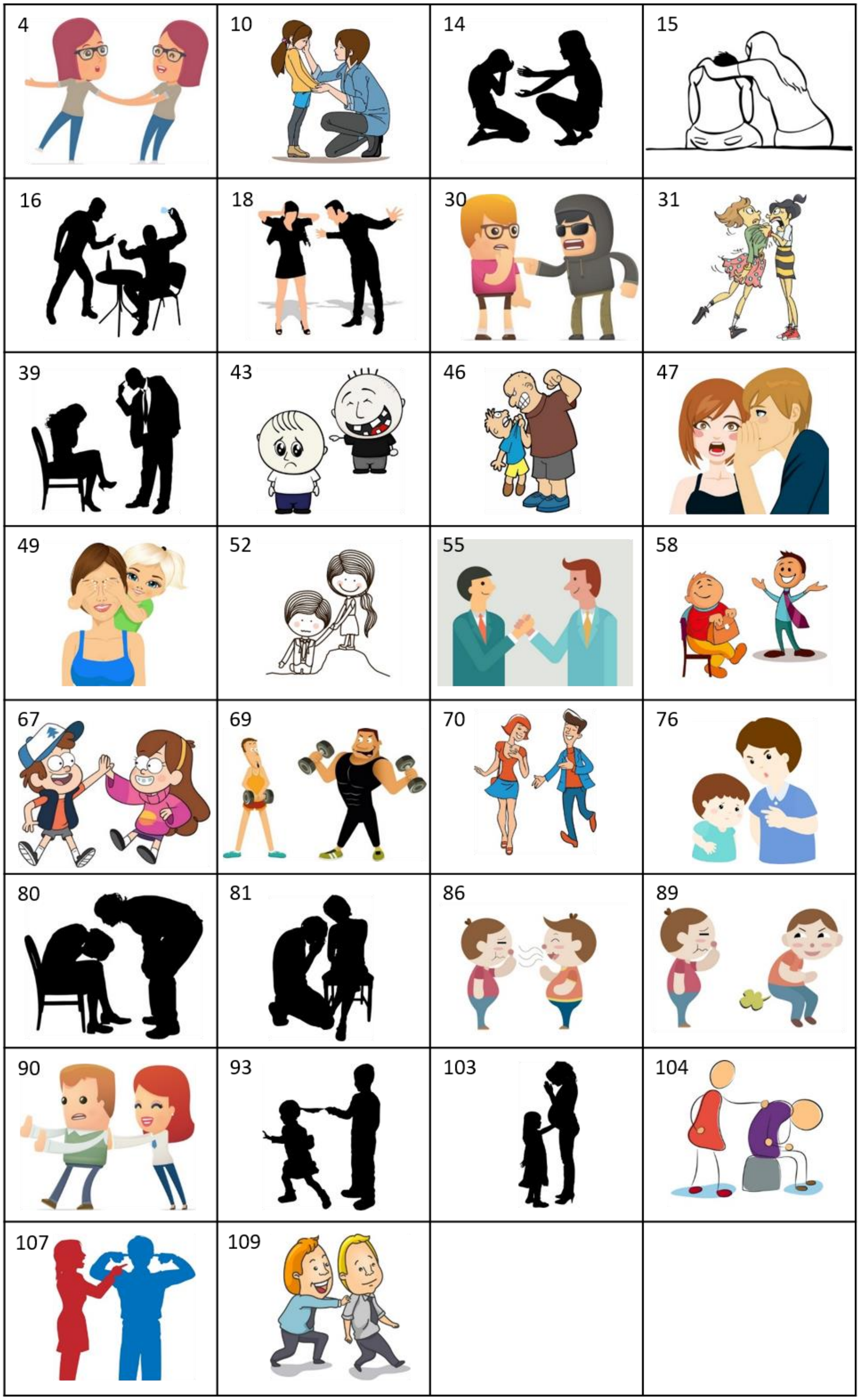

Figure B – All 30 images used for surveys. For survey 2, two variations of each figure were created, with blue arrow above one of the characters in each figure. Figures #103, #104 and #109 were removed from the analysis since the person performing the DA is on the left instead on the right. *Cartoons reprinted from Shutterstock.com under a CC BY license, with permission from Shutterstock.*

C. Screenshot of the survey 1

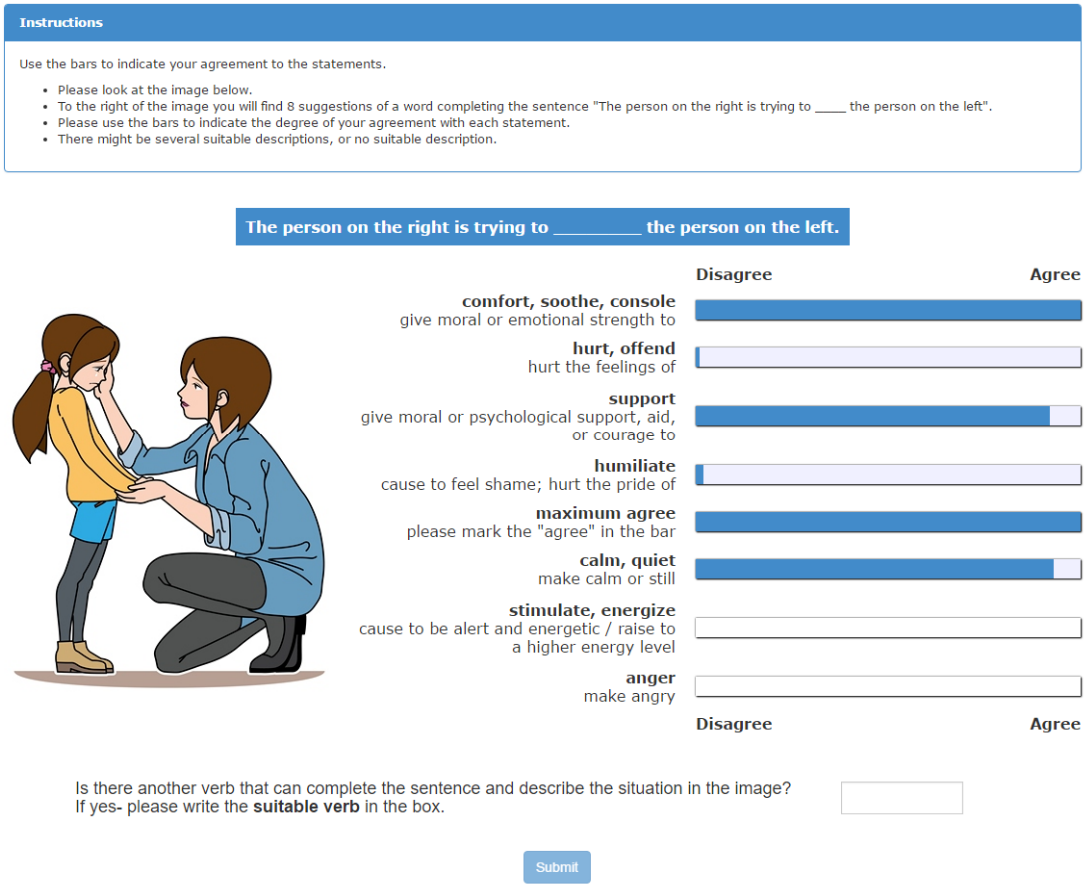

Cartoon reprinted from Shutterstock.com under a CC BY license, with permission from Shutterstock.

Figure C – Screenshot of survey 1 (DA words).

D. Screenshot of the survey 2

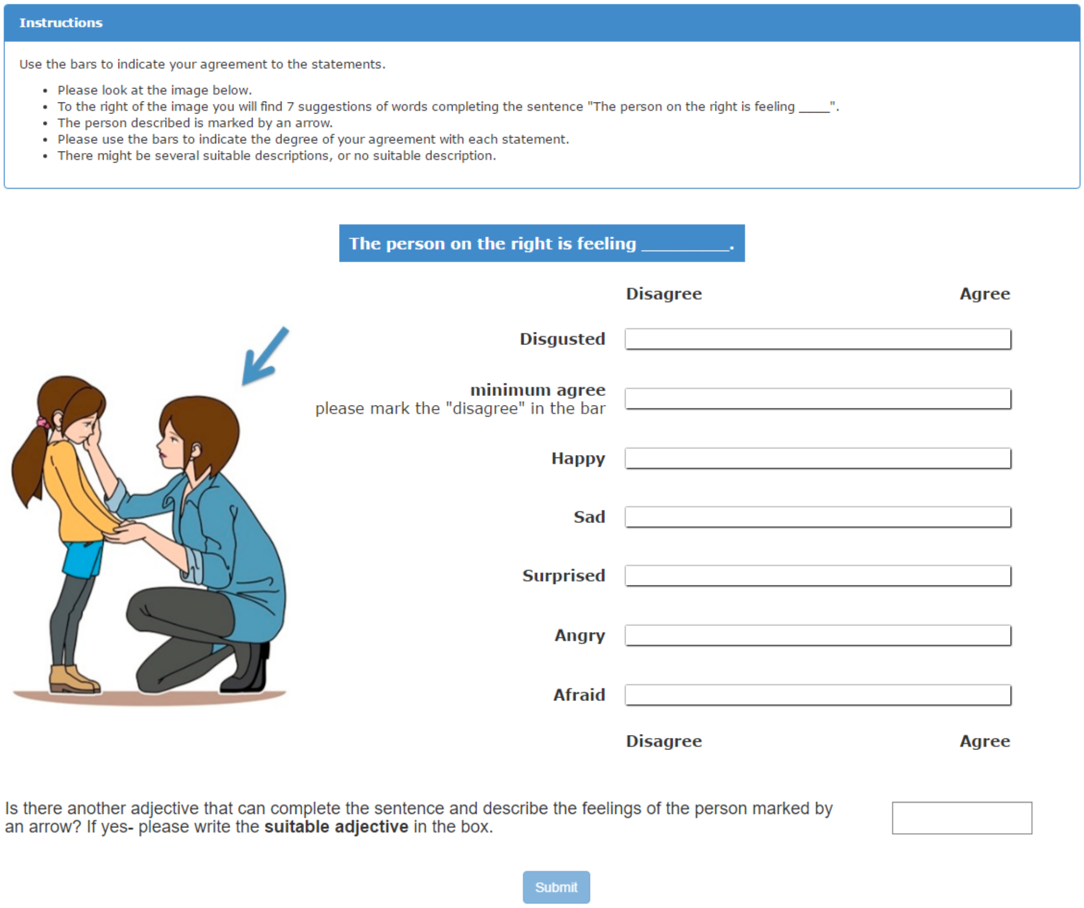

Instructions

Use the bars to indicate your agreement to the statements.

- Please look at the image below.
- To the right of the image you will find 7 suggestions of words completing the sentence "The person on the right is feeling ____".
- The person described is marked by an arrow.
- Please use the bars to indicate the degree of your agreement with each statement.
- There might be several suitable descriptions, or no suitable description.

The person on the right is feeling _________.

Disagree | Agree

Disgusted

minimum agree
please mark the "disagree" in the bar

Happy

Sad

Surprised

Angry

Afraid

Disagree | Agree

Is there another adjective that can complete the sentence and describe the feelings of the person marked by an arrow? If yes- please write the **suitable adjective** in the box.

Submit

Cartoon reprinted from Shutterstock.com under a CC BY license, with permission from Shutterstock.

Figure D – Screenshot of survey 2 (emotions).

E. Distribution of all answers of survey 1 (DA)

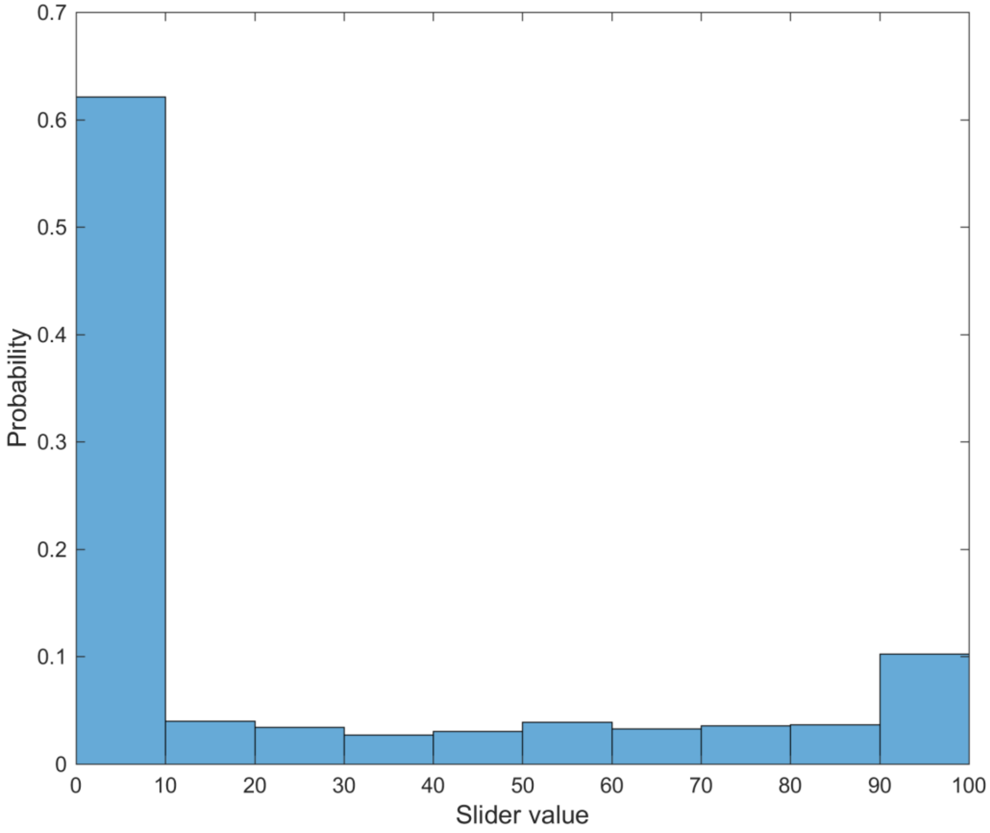

Figure E – Distribution of all answers of survey 1. The responses to the survey questions tended to be dichotomous. The distribution of all answers in experiment 1 is shown across the different deciles. Majority (62%) of answers were in the first decile corresponding to strong disagreement with the suggested verb. The distribution is bi-modal with the second peak at the 90-100 decile with about 10% of the answers. P-value < 10^{-4}.

F. Criteria to determine agreement is based on area under the cumulative distribution curve

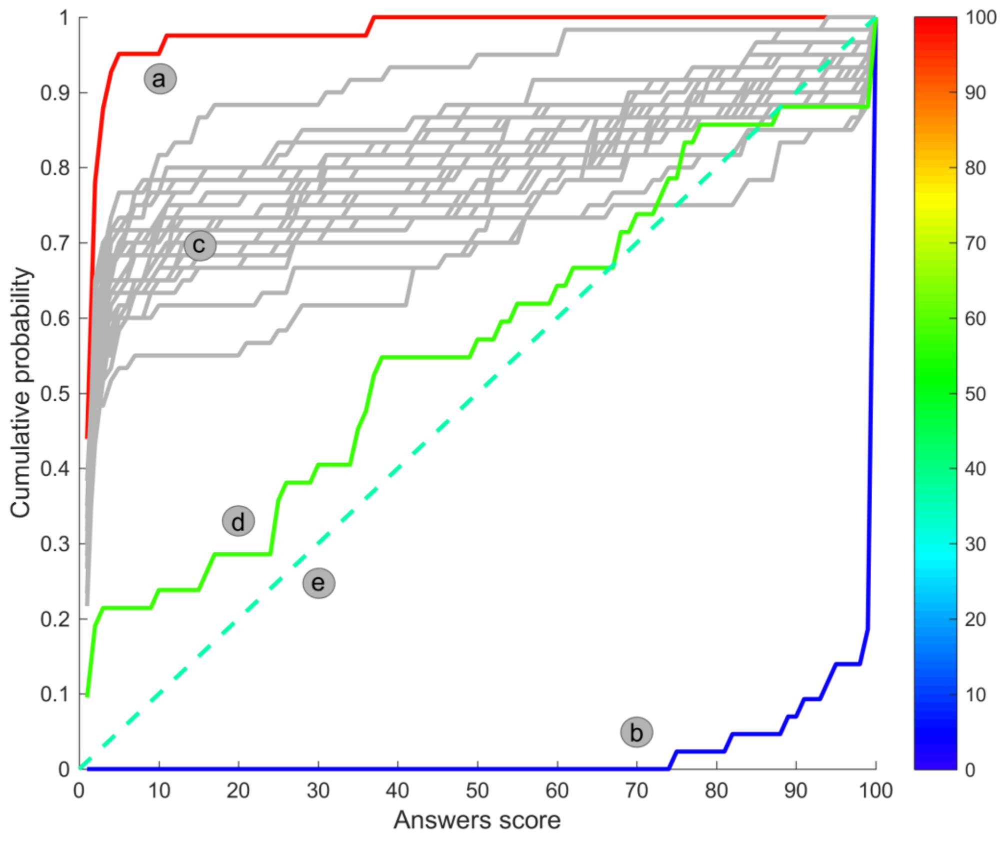

Figure F – Criteria to determine agreement is based on area under the cumulative distribution curve. Each line represents the empirical cumulative distribution function of answers to one question. The colors represent the area under the cumulative distribution (AUC). (a) Strong agreement on low score. (b) Strong agreement on high score. (c) 25 examples of randomly simulated responses. (d) Weak agreement on high score that is excluded by comparison to uniform distribution (e).

G. Cumulative distribution of all high agreement questions of survey 1

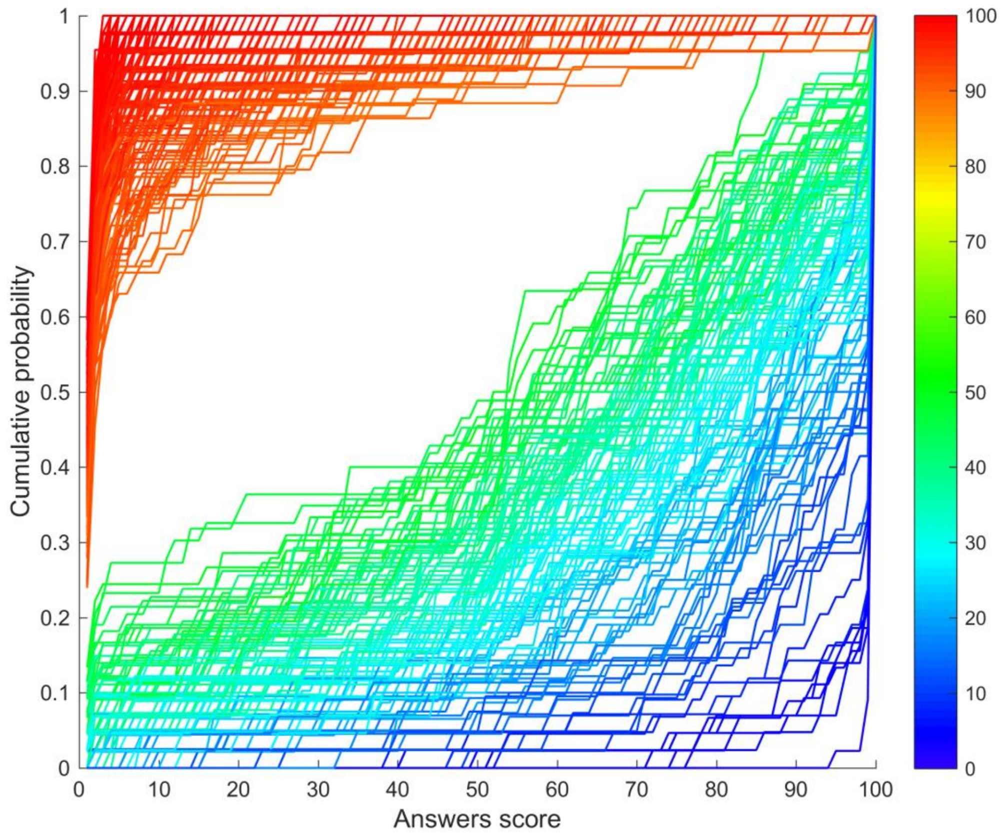

Figure G – Cumulative distributions of all high-agreement question of survey 1. The colors represent the area under the cumulative distribution line. The blue-green group of 131 lines is consisted of questions which passed the criteria for "agreement on high score". . The red group of 274 are for questions that passed the criteria for "agreement on low scores". In total, there was significant agreement in 71% out of 572 questions.

H. Distribution of all answers of survey 2 (emotions)

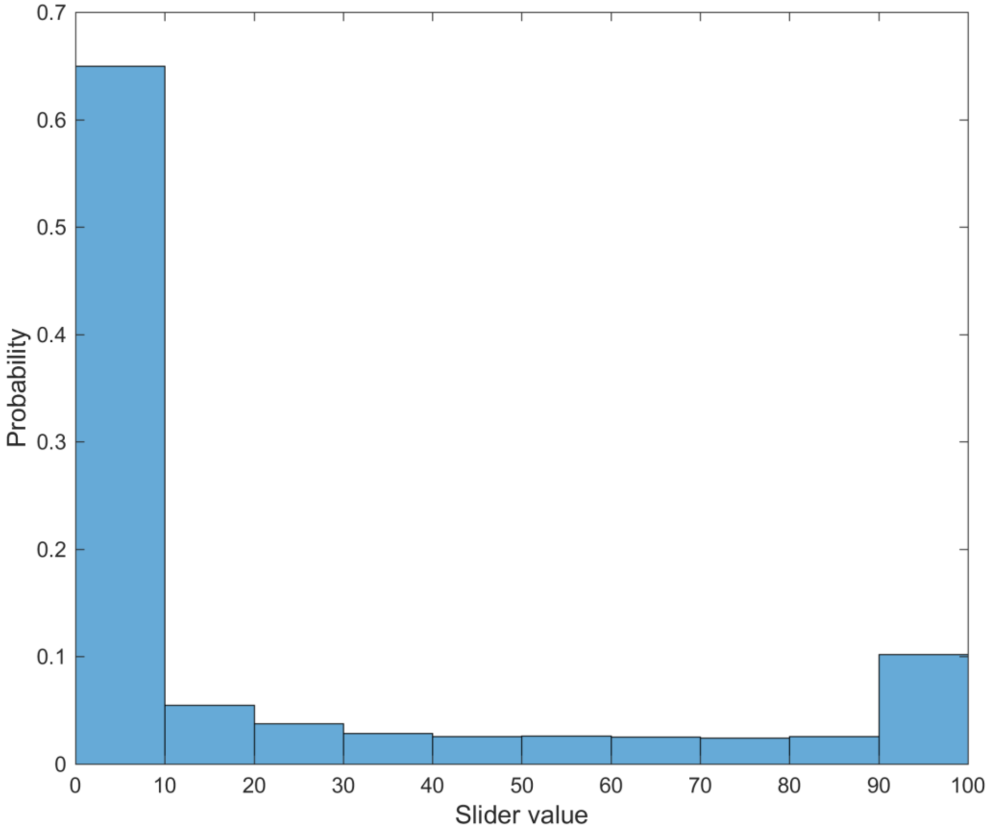

Figure H – Distribution of all answers of survey 2. The responses to the second survey questions show the same dichotomous distribution as the responses to the first survey. The distribution of all answers in experiment 2 is shown across the different deciles. Majority of answers were in the first decile, about 65%. The distribution is bi-modal with the second peak at the 90-100 decile with about 10% of the answers. P-value < 10-4.

I. Cumulative distribution of all high agreement questions of survey 2

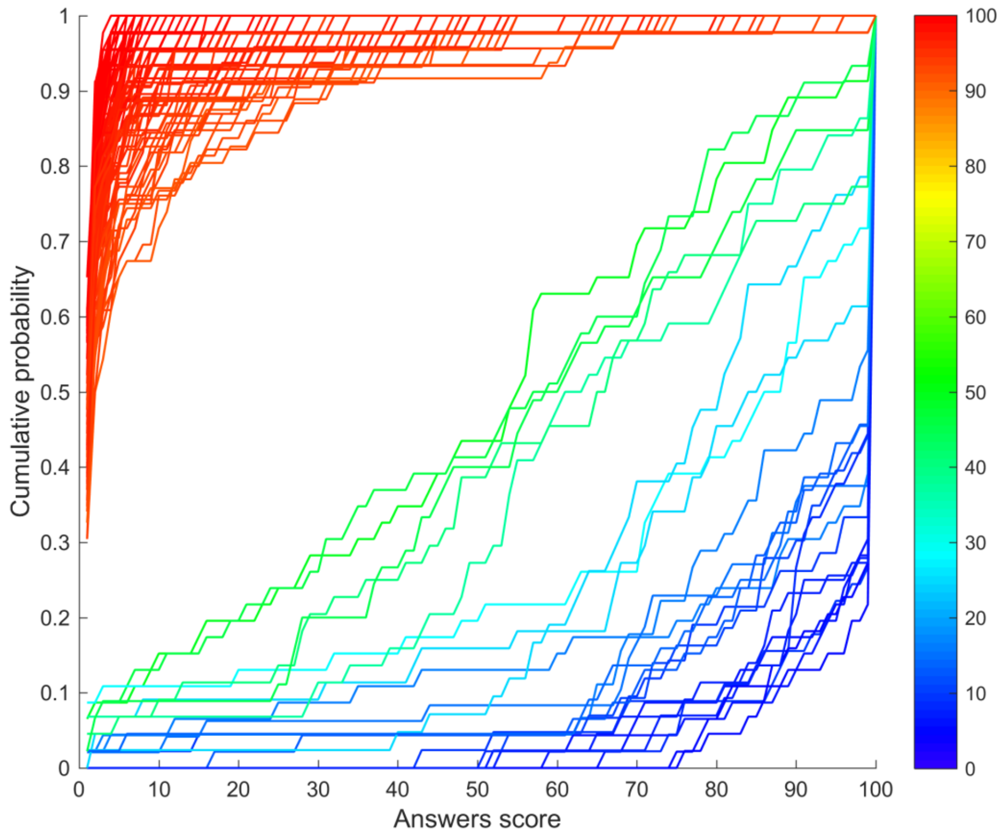

Figure I – Cumulative distributions of all high-agreement questions, asking about the emotion of the character who performs the DA. The colors represent the area under the cumulative distribution line. The blue-green group of 23 lines consists of questions with high-positive agreement. The red group of 85 lines shows the cumulative distribution of questions with high-negative agreement. In total, there was strong agreement in 78% of 162 questions.

Tables

Table A – List B – 150 DA words

support	1177772	shame	35598	allure	6903
please	957803	exhaust	35049	disgust	6548
hurt	599098	tease	34985	sicken	6248
engage	514981	pressure	34375	invigorate	6078
encourage	473843	delight	33556	fright	5984
bore	420284	appease	32055	engross	5464
worry	410222	annoy	31529	dishonor	5297
attack	292508	alert	29701	dishonour	4703
blame	283140	embarrass	28362	pester	4568
attract	262016	quiet	28234	solace	4288
capture	240000	lure	27920	enchant	3758
strengthen	211848	shock	25286	affront	3744
warn	149528	seduce	25170	sadden	3375
relax	144503	alarm	25004	joy	3361
stimulate	141912	intimidate	24710	enrage	3133
entertain	136524	entice	24286	astound	2781
inspire	131848	recreate	22000	swagger	2748
threaten	131438	harass	21624	embolden	2736
dominate	128239	scold	20952	abase	2658
impress	127656	startle	20299	browbeat	2527
absorb	119985	incite	20139	appal	2274
rush	115484	irritate	20116	hearten	1944
urge	114099	aggravate	19391	assault	1876
excite	104313	tire	18619	domineer	1855
ease	101472	rebuke	18438	affright	1739
hurry	97837	assail	18306	entrance	1706
concern	97565	astonish	17692	hassle	1592
comfort	93205	caution	16937	quieten	1549
surprise	82111	disgrace	16383	incense	1481
provoke	81963	pacify	15529	transfix	1426
awaken	80624	humiliate	14860	appall	1381
calm	73811	waken	13965	anger	1380
amuse	72503	befriend	13576	revolt	1294
discourage	72320	brace	12571	badger	1253
offend	71365	enliven	12549	exhilarate	1139
interest	69678	admonish	12030	humble	1054
cheer	68995	placate	11605	energise	951
arouse	64325	beguile	11246	elate	821
motivate	63288	reprove	11183	bug	741
confuse	62695	bully	10755	abash	724
frighten	60100	amaze	10439	tranquillise	559
rouse	52601	hush	10066	diss	538
tempt	52569	still	9932	trance	436
distract	51996	lull	9107	weary	401

scare	51869	belittle	8853	charm	366
squeeze	51644	fascinate	8792	spite	276
soothe	48386	unwind	8515	reproof	241
console	46132	energize	8492	nettle	198
mock	41182	gladden	8113	enamour	162
repel	40715	fatigue	7624	bullyrag	108
insult	37545	captivate	7301	enamor	65

Table A - List B – 150 DA words

The numbers next to the DA words represent their frequency according to the number of occurrence in the Google NGram database. The words in the list are presented in a descending order of frequencies.

Table B – Analysis of the Specific Affect Coding System (SPAFF) from Ref (1)

SPAFF code Gottman et al 2007	Function (SPAFF Manual)	Goal	Tactics		State
			Analysis	Dramatic Actions	
Affection	Expresses genuine caring and concern and offers comfort.		Elicit positive emotions<br>Decrease sadness, fear<br>Increase status	To support, to encourage, to comfort, to soothe, to calm	
Anger	Response to perceived violations<br>of the speaker's rights to autonomy and respect				Emotion - Anger
Belligerence	To "get a rise" out of the receiver through provocation of anger. The belligerent speaker is, in a sense, looking for a fight.		Elicit anger<br>Increase arousal	To anger, to irritate<br><br>As a threat: to threaten, to intimidate	
Contempt	To belittle, hurt, or humiliate. Communicates an icy lack of respect, often cruelty.		Elicit sadness, fear<br>lower other's status	To belittle, to hurt, to humiliate, to insult, to bully, to shame	
Criticism	Attack on someone's character or personality in a way that is not obviously insulting, as in contempt.<br>It is often accompanied by blame	Change behavior of other. It can be accompanied by several DAs that are used as a tactic to achieve this goal.	(i) Elicit guilt<br><br>(ii) Elicit fear<br><br>(iii) Elicit anger	(i) To scold, to blame<br><br>(ii) To threaten<br><br>(iii) To anger	
Defensiveness	Defensiveness functions to deflect responsibility or blame. It communicates a kind of innocent victimhood or righteous indignation.	Deflect responsibility.	(i) Decrease own status<br><br>(ii) Increase own status and lower other's (counterattack)	(i) To beg, to please, to charm<br><br>(ii) To threaten, to hurt, to belittle	
Disgust	Involuntary verbal or nonverbal reaction to a stimulus that is perceived to be noxious		When includes active responses such as mockery, insults or belittlement it		Emotion - Disgust

			is coded as **contempt**.		
Domineering	To exert and demonstrate control over one’s partner or a conversation		Lower other's status<br>Increase own status	To dominate, to belittle, to threaten, to bully	
Enthusiasm	Express a passionate interest in a person or activity …<br>Enthusiasm is infectious and often sudden, loud, boisterous, and energetic		Elicit happiness<br>Increase arousal of both.	To cheer, to uplift, to stimulate, to energize	
Fear/Tension	Communicates, usually involuntarily, fear, worry, anxiety, nervous anticipation, or dread.				Emotion - Fear
Humor	To share in mutual amusement and joy following a mutually recognized moment of absurdity or fun.		Elicit happiness, amusement<br>Increase other's arousal<br>Increase togetherness	To amuse, to uplift	
Interest	To communicate genuine interest in one’s partner through active elaboration or clarification seeking	Collect information and create good communication.	Elicit positive emotion<br>Increase other's status	To empower, to encourage	
Sadness	Behaviors that communicate loss, resignation, helplessness, pessimism, hopelessness, or a plaintive or poignant quiescence.				Emotion - Sadness
Stonewalling	Communicate an unwillingness to listen or respond to the receiver.<br>Communicating the message, “I’d rather not be here right now, and I don’t want to listen to you.”		Elicit negative emotion.<br>Lower other's status<br>Raise other's arousal	To anger, to belittle, to humiliate	

Threats	Particularly hostile form of domineering behavior in that their function is to control the behavior of the receiver by setting explicit conditions under which the receiver will be punished for behaving in ways the speaker finds undesirable.	Change behavior of other	Can be accompanied by various DAs	E.g. to threat, to hurt, to belittle, to impress	
Validation	Communicate sincere understanding and acceptance of one’s partner or of one’s partner’s views and opinions		Elicit positive emotion Increase other's status	To support, to encourage	
Whining	Make what might otherwise be an ordinary complaint into a plaintive or pleading form of emotional protest. Whining suggests an innocent victim stance		Decrease own status Can be accompanied by various DAs	E.g. to pressure, to blame, to urge	

Table B: Analysis of the Specific Affect Coding System (SPAFF) from Ref (1)**.**

The Specific Affect Coding System (SPAFF) was introduced by Gottman and Krokoff(2) in 1989 for the purpose of systematically observing affective behavior in the context of marital conflict. The SPAFF has been widely used in scientific research, and was refined over the years. This analysis, done by the authors, is based on the complete description of the SPAFF, including its history and coding manual, published in Ref (1).

Table C – Analysis of the FAU emotion corpus annotations from Ref (3)

FAU-AIBO annotation Batliner et al 2004	Description	Goal	Dramatic action (tactics)		State
			**Analysis**	**Actions**	
Joyful	The child enjoys AIBO's action and/or notices that something is funny.				Emotion – happy
Surprised	The child is (positively) surprised because obviously, he/she did not expect AIBO to react that way.				Emotion – positive surprise
Motherese	The child addressed AIBO in the way mothers/parents address their babies (also called 'infant-directed speech')—either because AIBO is well-behaving or because the child wants AIBO to obey; this is the positive equivalent to *reprimanding*.	Wants AIBO to OBEY- preserve or improve	Elicit positive emotion Increase other's arousal	To encourage, to soothe	
Bored	The child is (momentarily) not interested in the interaction with AIBO.				State – low arousal Can be related to sadness.
Emphatic	The child speaks in a pronounced, accentuated, sometimes hyper-articulated way but without 'showing any emotion'.		Increase other's arousal*	To urge, to rush	
Helpless	The child is hesitant, seems not to know what to tell AIBO next; can be marked by disfluencies and/or filled pauses.				State – anxiety
Touchy (irritated)	The child is slightly irritated; this is a pre-stage of anger.				State – low intense anger
Reprimanding	The child is reproachful, reprimanding, 'wags the finger'; this is the	Wants AIBO to OBEY- improve	Elicit negative emotion	To criticize, to scold	

	negative equivalent to *motherese*.		Increase other's arousal		
Angry	The child is clearly angry, annoyed, speaks in a loud voice.				emotion – anger

Table C: Analysis of the FAU emotion corpus annotations from Ref (3)
The FAU AIBO Emotion Corpus was created in order to develop and assess emotion recognition algorithms. It is based on recordings of children instructing a robotic dog to fulfil specific tasks.

*: based on authors' analysis of the recordings kindly provided by Steidl et al.

Table D – Analysis of ratings used in surgeons' voice-tone rating from Ref (4)

Voice ratings Ambady et al 2002	Definition (WordNet)	Goal	Dramatic action (tactics)		State
			**Analysis**	**Actions**	
Warm	Friendly and responsive	Make the patient feel comfortable	Elicit positive emotion	To encourage, to support	
Anxious/concerned	Feeling or showing worry or solicitude	Show concern for the patient	Elicit positive emotion	To support, to soothe	
Interested	Having or showing interest	Collect information and create good communication.	Elicit positive emotion Increase other's status	To empower, to encourage	
Hostile	Characterized by enmity or ill will		Elicit fear, sadness	To hurt, to offend, to humiliate, to upset	
Sympathetic	Expressing or feeling or resulting from sympathy or compassion or friendly fellow feeling	Make the patient feel a friendly environment	Elicit positive emotion Lower arousal	To comfort, to soothe, to console, to calm, to quiet	
Professional	Characteristic of or befitting a profession	Open to interpretation. E.g.<br>• Make the patient feel secure<br>• Keep the interaction not personal	(i) Increase security Increase own status<br><br>(ii) Decrease relationship	(i) To impress, to calm<br><br>(ii) To distance*	
Competent	Properly or sufficiently qualified or capable or efficient	Make the patient feel secure	Increase security Increase own status	To impress, to calm	
Dominant	Exercising influence or control	Control the interaction	Lowers other's status Increase own status	To dominate, to belittle, to threaten, to bully<br>To impress	
Satisfied	Filled with satisfaction				State - satisfaction
Genuine	Not pretended;				

	sincerely felt or expressed				

Table D: Analysis of ratings used in surgeons' voice-tone rating from Ref (4)

In this study of doctor-patient interaction, surgeons were audiotaped while speaking to their patients during office visits. Very brief samples (10-seconds) of the conversations were rated by coders. As the raters were not given any further training or any feedback, we use here the WordNet definition for the voice ratings, choosing the definition most appropriate for the doctor-patient context.

*To distance may not be the best verb to describe this DA, as it can also be perceived as a goal (i.e. make the interaction less personal). As a DA, we refer to an action done by the doctor in order to deflate a patient's attempt to bring the interaction to an emotional or personal level. This could be regarded as a version of Gottman's stonewalling.

List of Appendices

A. Collection of Dramatic action (DA) lists

In order to narrow down list A of 3602 transitive verbs to a concise list of DAs, we used previous lists of DA words from various non-scientific sources on the web. The main lists are available on our website here: link

B. Criteria for inter-rater agreement

To compute agreement for each question (image-DA combination), one cannot use Krippendorff's alpha because it requires comparison between at least two questions (8). We therefore used a statistical test that picked up on the fact that, for the vast majority of questions, most respondents agreed on either high or low scores. The challenge was that the responses had a noisy structure: often a sizable minority of the respondents had a wide range of responses. To address this noise, we used a statistical test based on bootstrapping (9). For each question, we listed the respondents that answered (N=39-47 in the dataset filtered using the attention checks). We generated N shuffled responses, by choosing for each respondent a response score taken at random from the set of all responses of that responder in the survey, thus keeping the statistics of each respondent the same. We aimed to score as "agreement on high score" a situation where the majority of responders agree on very high scores. We could not use median or mean score because the noise in the responses could show a relatively high median or mean but with a flat distribution of answers. We therefore evaluated the cumulative distribution of responses for the shuffled data, and computed the area under this cumulative distribution, denoted AUC (area under the curve). The AUC is 0 if all respondents answered the maximal score (100), and is 1 if all respondents answered 0, and is a sensitive discriminator against uniform distributions of scores (AUC=0.5). We repeated the shuffling 10^4 times for each question, and thus generated an ensemble of 10^4 shuffled AUCs for each question. We scored a question as "agreement on high score" if the AUC of the real data was in the lowest (5/M)%=0.0023% of the shuffled data, where M=22 is the number of words tested for that image (Figure F-a). Dividing by M is a Bonferroni correction for multiple hypothesis testing for each image. Similarly, we scored a question as "agreement on low score" if the AUC of the real data was in the highest (5/M)% of the shuffled data (Figure F-b). Figure F-c shows a subset of the 10000 shuffled datasets used to determine significance.

Our data was generally skewed towards low scores (See SI Figure G). As a result, questions such as depicted in Figure F-d are scored as “agreement on high-score” with a significantly low p-value, despite the fact that the majority of responders to this question scored low (more than 50% of responders gave score < 50). In order to filter out such cases (which amount to about 7% of the questions), we added an additional criterion for “agreement on high-score”, namely that the AUC is smaller than the AUC of a uniform distribution of scores (i.e. AUC< 0.5 for positive agreement).

References

1. Coan JA, Gottman JM (2007) The Specific Affect Coding System (SPAFF). *Handbook of Emotion Elicitation and Assessment*, eds Coan JA, Allen JJB (Oxford University Press), pp 267–285. 1st editio.

2. Gottman JM, Krokoff LJ (1989) Marital interaction and satisfaction: A longitudinal view. *J Consult Clin Psychol* 57(1):47–52.

3. Batliner A, Steidl S, Hacker C, Nöth E (2008) Private emotions versus social interaction: a data-driven approach towards analysing emotion in speech. *User Model User-adapt Interact* 18(1–2):175–206.

4. Ambady N, et al. (2002) Surgeons' tone of voice: A clue to malpractice history. *Surgery* 132(1):5–9.

5. Park S (2013) Persuasiveness in social multimedia. *Proceedings of the 15th ACM on International Conference on Multimodal Interaction - ICMI '13* (ACM Press, New York, New York, USA), pp 321–324.

6. Tosti-Kharas J, Conley C (2016) Coding Psychological Constructs in Text Using Mechanical Turk: A Reliable, Accurate, and Efficient Alternative. *Front Psychol* 7:741.

7. Cocos A, Masino A, Qian T, Pavlick E, Callison-Burch C (2015) Effectively Crowdsourcing Radiology Report Annotations. *Proceedings of the Sixth International Workshop on Health Text Mining and Information Analysis* (Association for Computational Linguistics, Stroudsburg, PA, USA), pp 109–114.

8. Krippendorff K *Content Analysis: An Introduction to Its Methodology* (SAGE Publications, Inc). 3rd Ed.

9. Efron B, Tibshirani RJ *An Introduction to the Bootstrap* (Chapman and Hall/CRC). 1st Ed.
