# Supplementary material for: Dramatic action: A theater-based paradigm for analyzing human interactions
Source: PLoS One. 2018 Mar 8;13(3):e0193404. doi: 10.1371/journal.pone.0193404 (PMC5843267; doi:10.1371/journal.pone.0193404)
Supplement: S1 Text — Information regarding the figures’ copyrights. (PDF) [file pone.0193404.s002.pdf]

The structure of influence tactics: a cartoon-based measurement system derived from the theater concept of dramatic action

Yuvalal Liron, Noa Raindel, Uri Alon

Figures Copyright Information

All cartoon images shown in the manuscript and supporting information were purchased from Shutterstock, stock media provider of royalty-free images.

Shutterstock's Terms of Service explicitly permits the usage of images in e-publications. An excerpt from the Shutterstock license is included below.

As the Shutterstock license permits a very wide range of usage, they do not provide explicit consent letters. Shutterstock costumer support have confirmed that the Shutterstock license allows publishing the images under the CC BY license. The reference to our query to Shutterstock support is **ref:_00D301GgSC._5000c1SXKwE:ref**.

Our group's Shutterstock ID: **153844018**

Our Order ID for the images in the manuscript: **SSTK-05FCE-EAEA**

An Excerpt from Shutterstock's Terms of Service

PART I – VISUAL CONTENT LICENSES

1. Shutterstock hereby grants you a non-exclusive, non-transferable right to use, modify and reproduce Visual Content worldwide, in perpetuity, as expressly permitted by the applicable license and subject to the limitations set forth herein:

   A. IMAGE LICENSES

      i. A STANDARD IMAGE LICENSE grants you the right to use Images:

         1. As a digital reproduction, including on websites, in online advertising, in social media, in mobile advertising, mobile "apps", software, e-cards, e-publications (e-books, e-magazines, blogs, etc.), email marketing and in online media (including on video-sharing services such as YouTube, Dailymotion, Vimeo, etc., subject to the budget limitations set forth in sub-paragraph I.a.i.4 below);

Full License: https://www.shutterstock.com/license

Credit attribution

The Images shown in the manusctipt were created by the following Shutterstock artists:

- Anabela88
- artenot
- Fairmacy
- Ficus777
- grmarc
- Ildar Galeev
- jesadaphorn
- John T Takai
- Kakigori Studio
- KrechOksana
- lena_eva
- Lorelyn Medina
- majivecka
- Marina BH
- Michael D Brown
- Onanong Tasanapitak
- Sangoiri
- Sergio Hayashi
- VasutinSergey
